# Genomic signatures associated with epidemiologically defined high-risk pathogenic *Escherichia coli* isolates identified by interpretable machine learning

**DOI:** 10.71150/jm.2604011

**Published:** 2026-08-31

**Authors:** Yoojung Hwang, Woo Young Cho, Woojung Lee, Insun Joo, Jeong-Ih Shin, Mi-Ran Seo, Seung-Hun Shin, Kwan Soo Ko, Kun Taek Park, Yeun-Jun Chung, Seung-Hyun Jung

**Affiliations:** 1 Department of Medical Sciences, Graduate School of The Catholic University of Korea, Seoul 06591, Republic of Korea; 2 Catholic Research Institute for Human Genome Polymorphism, College of Medicine, The Catholic University of Korea, Seoul 06591, Republic of Korea; 3 ConnectaGen, Hanam 12918, Republic of Korea; 4 Division of Food Microbiology, National Institute of Food and Drug Safety Evaluation, Ministry of Food and Drug Safety, Cheongju 28159, Republic of Korea; 5 Department of Microbiology, Sungkyunkwan University School of Medicine, Suwon 16419, Republic of Korea; 6 Department of Biological Sciences, Inje University, Gimhae 50834, Republic of Korea; 7 Department of Microbiology, College of Medicine, The Catholic University of Korea, Seoul 06591, Republic of Korea; 8 Department of Biochemistry, College of Medicine, The Catholic University of Korea, Seoul 06591, Republic of Korea

**Keywords:** *Escherichia coli*, genomic risk prioritization, whole-genome sequencing, machine learning, foodborne outbreak

## Abstract

Pathogenic *Escherichia coli* is a major cause of foodborne illness worldwide and includes strains capable of causing severe disease. To establish a genome-informed framework for foodborne outbreak surveillance, we analyzed 1,029 *E. coli* isolates from clinical, food, livestock, and environmental sources using whole-genome sequencing. Pathogenic isolates obtained from human clinical cases or linked to documented outbreaks were classified as epidemiologically defined high-risk (EpiHR), whereas the remaining pathogenic isolates were classified as non-EpiHR. Virulence-associated genomic features were extracted using a bioinformatics pipeline, and four machine learning (ML) algorithms, including gradient boosting machine, random forest (RF), and support vector machines with linear and radial basis function kernels, were evaluated. Among them, the RF model showed the best performance, achieving an area under the curve (AUC) of 0.98 and accuracy of 0.93 in 10-fold cross-validation. Additional leave-one-group-out validation showed retained discrimination across held-out sequence types and serotypes, although performance was reduced when isolates were grouped by isolation source. Evaluation using an independent test dataset of 1,908 publicly available pathogenic *E. coli* genomes showed an AUC of 0.97 and a sensitivity of 0.98. Feature importance analysis using Shapley additive explanations identified influential predictive features, including *traT*, *etpB*, and enterotoxin-associated genes. A reduced 10-feature model achieved an AUC of 0.79 in the independent test dataset, supporting its exploratory use for future simplified screening approaches. These results indicate that genome-based ML provides a sensitive framework for surveillance-oriented prioritization of EpiHR pathogenic *E. coli* isolates, with model predictions interpreted together with epidemiological information.

## Introduction

Although *Escherichia coli* (*E. coli*) is a common commensal of the gastrointestinal tract of humans and animals, certain strains cause intestinal and extraintestinal diseases (Kaper et al., 2004). Pathogenic *E. coli* remains a major cause of foodborne outbreaks. In South Korea, 46 outbreaks involving 2,287 patients were attributed to pathogenic *E. coli* in 2024, accounting for 26.0% of all foodborne illness cases. Notable outbreaks include O157:H7 in Washington State in 1993, which resulted in 501 reported cases and three confirmed deaths (Bell et al., 1994), and O104:H4 in Germany in 2011, which caused more than 3,800 reported cases, including 834 cases of hemolytic-uremic syndrome (Frank et al., 2011). Other O-serotypes, including O25, O26, O111, and O121, have also been associated with foodborne outbreaks (Dewey-Mattia et al., 2018; Weerdenburg et al., 2023; Yang et al., 2017), highlighting the need for approaches that prioritize outbreak-associated risk beyond conventional serotyping.

A pathotype is defined as a pathogenic variant within a taxonomically related group of microorganisms that typically colonize a host asymptomatically (Riley, 2020). Diarrheagenic *E. coli* is primarily categorized into six pathotypes based on specific virulence genes (Croxen and Finlay, 2010; Robins-Browne et al., 2016): *elt* and *est* for enterotoxigenic *E. coli* (ETEC); *eae* and *bfpA* for enteropathogenic *E. coli* (EPEC); *stx1* and *stx2* for Shiga toxin–producing *E. coli* (STEC); *ipaH* and *invE* for enteroinvasive *E. coli* (EIEC); *aggR* for enteroaggregative *E. coli* (EAEC); and Afa/Dr adhesins for diffusely adhering *E. coli* (DAEC). This scheme is useful for outbreak identification, tracking, and clinical interpretation, but may be insufficient because of the evolutionary plasticity of *E. coli* (Robins-Browne et al., 2016). For example, EPEC strains harboring heat-labile enterotoxin genes characteristic of ETEC and ETEC strains expressing Shiga toxin have been reported (Dutta et al., 2015; Zhang et al., 2007). Moreover, genomic factors beyond canonical pathotype-defining markers, including those involved in colonization, secretion systems, and capsule formation, may contribute to clinical or outbreak-associated phenotypes (Ohya et al., 2025). Thus, genome-based strategies could improve prioritization of pathogenic *E. coli* isolates associated with clinical cases or documented outbreaks.

Whole-genome sequencing (WGS) enables molecular typing and virulence profiling of pathogens, thereby strengthening routine foodborne disease surveillance, outbreak detection, and source identification. Accordingly, the World Health Organization released guidelines on the use of WGS for foodborne disease surveillance in 2023. Machine learning (ML) methods can identify predictive patterns in high-dimensional WGS data (Vorimore et al., 2023) and have been used to predict host specificity, zoonotic potential, and specific types of STEC (Im et al., 2021; Lupolova et al., 2016, 2017; Vorimore et al., 2023). For example, a previous study developed a support vector machine (SVM) model for STEC assessment that successfully identified isolates derived from major outbreak sources (Im et al., 2021). However, a genome-informed framework for prioritizing epidemiologically defined high-risk (EpiHR) pathogenic *E. coli* isolates across diverse pathotypes has not yet been established.

This study aimed to establish a genome-informed framework for prioritizing EpiHR pathogenic *E. coli* isolates in foodborne outbreak surveillance. We analyzed virulence-associated genomic features from 1,029 *E. coli* isolates collected in South Korea using WGS and developed an interpretable ML-based risk-prioritization model. The model was further evaluated using an independent test dataset comprising 1,908 publicly available pathogenic *E. coli* genomes.

## Materials and Methods

### WGS and bioinformatic analysis

A total of 1,029 *E. coli* isolates collected nationwide during the second phase of a foodborne pathogen surveillance study conducted in South Korea were included in the analysis. Details of the isolates, including their sources, are provided in Table S1. Genomic DNA was extracted using the iDetect gDNA Prep Kit for Microbes (ConnectaGen, Korea), and DNA concentrations were measured using a Qubit 2.0 Fluorometer (Thermo Fisher Scientific, USA). Sequencing libraries were prepared and processed as previously described (Hong et al., 2025; Shin et al., 2025). Briefly, paired-end libraries were prepared using the TruSeq Nano DNA sample preparation kit (Illumina, USA) and sequenced on an Illumina NextSeq platform.

Raw sequencing reads were assembled using SPAdes (Prjibelski et al., 2020). Assembly quality was assessed using QUAST (Mikheenko et al., 2023), and assemblies with total lengths outside the range of 4–6 Mb were excluded. Taxonomic profiling was performed using Kraken2 and Bracken to exclude assemblies with < 90% *E. coli* abundance or ≥ 5% abundance of other species (Lu et al., 2022). *E. coli* pathotypes were determined by BLASTN searches against pathotype-specific virulence genes using identity and coverage thresholds of ≥ 80% and ≥ 60%, respectively (Robins-Browne et al., 2016). Multi-locus sequence typing was performed using the MLST tool (https://github.com/tseemann/mlst) based on Achtman’s scheme from the PubMLST database (Jolley and Maiden, 2010). Serotypes were predicted using SerotypeFinder (Joensen et al., 2015). Virulence-associated genes were identified using ABRicate (v1.0.1) with a custom-built database primarily derived from the Virulence Factor Database (Liu et al., 2019), applying identity and coverage thresholds of ≥ 90% and ≥ 60%, respectively. A maximum-likelihood phylogenetic tree based on core gene single-nucleotide polymorphisms was constructed using IQ-TREE2 (Minh et al., 2020). The *E. coli* K-12 reference genome (GenBank accession: NC_000913) was used as an outgroup to root the tree, which was visualized using Microreact (Argimon et al., 2016). Functional annotation was performed using Prodigal (Hyatt et al., 2010) and eggNOG-mapper (Cantalapiedra et al., 2021). This study was reviewed and deemed exempt by the Institutional Review Board of the Catholic University of Korea, College of Medicine (approval number: MC24SASI0019).

### Machine learning models

ML-based risk-prioritization models were developed to distinguish EpiHR isolates from non-EpiHR isolates. EpiHR isolates were defined as pathogenic isolates obtained from human clinical cases or linked to documented outbreaks regardless of source, whereas non-EpiHR isolates were pathogenic isolates that did not meet these criteria. Isolates lacking pathotype-defining genes detected by WGS were classified as pathotype-negative and excluded from model development (Croxen and Finlay, 2010; Robins-Browne et al., 2016).

Virulence-associated genes identified using ABRicate and confirmed by BLASTN were used as candidate predictors. Genes with identical names were consolidated into a single feature regardless of accession number, generating a binary presence/absence matrix in which 1 indicated gene presence and 0 indicated gene absence. The total number of detected virulence-associated genes in each isolate was included as an additional feature.

Four classification algorithms were evaluated: gradient boosting machine (GBM), random forest (RF), and SVMs with linear and radial basis function kernels. Model performance was assessed using both 5-fold and 10-fold cross-validation (CV) based on pooled out-of-fold predictions, with EpiHR isolates designated as the positive class. Evaluation metrics included accuracy, precision, sensitivity, specificity, F1-score, area under the curve (AUC), and Matthews correlation coefficient (MCC) (Rainio et al., 2024). The model showing the highest AUC in CV was selected as the final model and retrained using the complete training dataset.

To assess potential performance inflation arising from group-specific genomic or epidemiological structure, additional leave-one-group-out (LOGO) validation was performed in the training dataset according to sequence type (ST), serotype, and isolation source. In each LOGO scheme, isolates belonging to one group were held out in turn, and the model was trained using isolates from all remaining groups. Performance was calculated from pooled predictions for the held-out isolates.

Feature reduction was evaluated within the training dataset using a 10-fold CV procedure designed to avoid information leakage. In each fold, Shapley additive explanations (SHAP)-based feature ranking was derived exclusively from the training portion, and RF models based on the top 5 to 50 gene-level features, added in increments of five, were evaluated in the corresponding held-out fold (n_estimators = 100, max_depth = 11, min_samples_split = 3, class_weight = 'balanced', and random_state = 58141). The overall virulence-associated gene burden was excluded from the reduced gene-level feature sets. A reduced feature-set size was selected considering CV performance and model parsimony. After determining the feature-set size, the final fixed gene-level features were identified by SHAP ranking using the complete training dataset, and an RF model trained with these features was evaluated in the independent test dataset. All model training and evaluation were performed using the scikit-learn package in Python. Model outputs were visualized using Matplotlib and Seaborn.

### Implementation of the classifier and independent test dataset evaluation

The classifier was implemented in Python. The pipeline accepts draft genome assemblies in FASTA format as input and performs four sequential steps: 1) BLASTN-based pathotyping, 2) ABRicate-based virulence-associated gene identification and summarization, 3) ML-based EpiHR probability estimation and classification, and 4) automated report generation (Fig. S1). The source code is publicly available on GitHub (https://github.com/IRCGP-Lab/EcoML-VP).

For independent test dataset evaluation, 1,908 publicly available pathogenic *E. coli* genomes with available epidemiological metadata were analyzed (Table S2). For comparison, all candidate models fitted using the complete training dataset were applied to the independent test dataset without model refitting or feature reselection. Using a default classification threshold of 0.5, performance was assessed using AUC, sensitivity, specificity, positive predictive value (PPV), negative predictive value (NPV), MCC, and accuracy.

To examine the practical effect of the classification threshold, threshold-dependent performance was evaluated in the independent test dataset across probability thresholds ranging from 0.1 to 0.9. Model calibration was assessed using a calibration curve comparing predicted probabilities with the observed frequency of EpiHR isolates. Performance was additionally stratified by isolation source, pathotype, serotype, and ST to evaluate heterogeneity across epidemiological and genomic subgroups.

## Results

### Genomic and pathotype profiles of *E. coli* isolates

A total of 1,029 *E. coli* isolates from diverse sources were analyzed, including 319 clinical (31.0%), 501 food-derived (48.7%), 80 livestock-derived (7.8%), and 129 environmental (12.5%) isolates (Fig. 1A). The analyzed assemblies had a median of 121 contigs per genome (range, 23–2,436) and a median genome size of 5.1 Mb (range, 4.5–5.9 Mb) (Table S1). MLST analysis identified 174 STs, of which ST752 was the most prevalent (n = 135, 13.1%), followed by ST2040 (n = 73, 7.1%) and ST10 (n = 47, 4.6%) (Fig. 1A). WGS-based serotyping identified 255 serotypes, with O159:H20 being the most prevalent (n = 73, 7.1%), followed by OUT (untypeable):H21 (n = 57, 5.5%) and O169:H41 (n = 36, 3.5%). O157:H7, a well-characterized serotype associated with EHEC (STEC/EPEC hybrid), was identified in 26 isolates (2.5%). Based on pathotype-defining genes, 140 isolates (13.6%) were classified as non-pathogenic, whereas 889 isolates (86.4%) were assigned to pathogenic pathotypes, including 345 EPEC (33.5%), 230 ETEC (22.4%), 190 STEC (18.5%), 55 EAEC (5.3%), 40 EHEC (3.9%), and 29 hybrid isolates excluding EHEC (2.8%).

A total of 370 virulence-associated genes were identified in the 1,029 isolates, with an average of 101.1 genes per isolate (range, 55–186). Virulence-associated gene counts varied across pathotypes and isolation sources (Fig. 1B). EHEC isolates harbored the highest number of virulence-associated genes (an average of 169.2), whereas ETEC isolates showed the lowest (an average of 77.2). Clinical EHEC isolates contained significantly more virulence-associated genes than food-derived EHEC isolates (*P* = 0.025; Mann–Whitney U test).

### Machine learning‑based prioritization of EpiHR pathogenic *E. coli* isolates

Among the 889 pathogenic isolates, 350 were categorized as EpiHR and 539 as non-EpiHR. The model input comprised 362 virulence-associated gene-level features and the total virulence-associated gene burden per isolate. All evaluated models showed high discriminatory performance, with AUC values exceeding 0.94 in cross-validation (Fig. 2A, Table 1). In 10-fold CV, the RF model achieved the highest AUC of 0.98, with an accuracy of 0.93, precision of 0.93, F1-score of 0.91, and MCC of 0.86, and was therefore selected for subsequent analyses. Accuracy exceeded 0.87 across all held-out folds of the 10-fold CV (Table S3). Using this model, 315 of 350 EpiHR isolates (90.0%) were predicted as EpiHR with probabilities ≥ 0.5, whereas 514 of 539 non-EpiHR isolates (95.4%) were predicted as non-EpiHR with probabilities < 0.5 (Fig. 2B). The average predicted probability was 0.86 for EpiHR isolates and 0.11 for non-EpiHR isolates, demonstrating clear separation between the two groups (Fig. 2C).

To evaluate the influence of group-specific genomic and epidemiological structure on model performance, additional LOGO validation analyses were conducted in the training dataset using ST, serotype, and isolation source as grouping variables (Table S4). Performance was lower than that observed in isolate-level CV but remained high in the ST- and serotype-based LOGO analyses, with AUC values of 0.94 and 0.93, respectively. Precision remained 0.93 and 0.91, and specificity was 0.96 in both analyses. In contrast, isolation source-based LOGO validation showed a greater reduction in performance, with an AUC of 0.79, accuracy of 0.74, and MCC of 0.46. These results indicate that the model retained discriminatory ability across held-out STs and serotypes, whereas isolation-source structure contributed more substantially to model performance.

### Patterns of predicted EpiHR probabilities across isolation sources and serotypes

Predicted EpiHR probabilities were examined according to isolation source and outbreak association. Consistent with their inclusion in the EpiHR group, most pathogenic clinical isolates were predicted as EpiHR (274/287, 95.5%), with an average predicted probability exceeding 0.90 (Fig. 3A). Among food-derived isolates, those not linked to outbreaks had a low average predicted probability of 0.11, with 19 of 423 isolates (4.5%) predicted as EpiHR, whereas outbreak-associated isolates showed a higher average probability of 0.65, with 35 of 52 isolates (67.3%) predicted as EpiHR. A similar pattern was observed for environmental isolates: non-outbreak isolates exhibited an average probability of 0.12 (6/67, 9.0% predicted as EpiHR), whereas outbreak-associated isolates had an average probability of 0.66 (6/11, 54.5% predicted as EpiHR). Livestock-derived isolates not linked to outbreaks showed consistently low predicted probabilities (average, 0.06), and none were predicted as EpiHR.

Regarding serotypes, 34 had an average predicted probability ≥ 0.5, whereas 77 had average probabilities < 0.5 (Fig. 3B). Notably, 19 serotypes exhibited an average predicted probability > 0.7, including O157, O44, O17, O6, O138, O159, O169, O181, and O176, several of which have previously been associated with foodborne outbreaks (Cho et al., 2014; Gross et al., 1976; Heiman et al., 2015; Hirose et al., 2023; Scheutz et al., 2004; Shin et al., 2016; Smith et al., 1994; Wang et al., 2005). In contrast, 33 serotypes, including O132, O186, O100, O116, O113, O168, O150, and O185, had average predicted probabilities < 0.1. These findings show that predicted EpiHR probabilities differed across isolation sources and serotypes, providing descriptive context for surveillance-oriented prioritization.

### Independent test dataset evaluation of the RF model

An independent test dataset comprising 1,908 pathogenic *E. coli* genomes was analyzed (Table S2). In contrast to the training dataset, this dataset consisted predominantly of environmental isolates, including 79 clinical (4.1%), 192 food-derived (10.1%), 95 livestock-derived (5.0%), and 1,542 environmental (80.8%) isolates. The assemblies had a median of 157 contigs per genome (range, 2–839) and a median genome size of 5.3 Mb (range, 4.7–6.0 Mb). According to the epidemiological criteria, 146 isolates were categorized as EpiHR and 1,762 as non-EpiHR. The EpiHR group was highly concentrated in ST11 (124/146, 84.9%) and O157:H7 (126/146, 86.3%).

Among the candidate models applied to the independent test dataset, the RF model showed the highest AUC of 0.97 (Fig. 4A). At the default EpiHR probability threshold of 0.5, the RF model yielded 144 true-positive, 2 false-negative, 442 false-positive, and 1,320 true-negative classifications, corresponding to a sensitivity of 0.98, specificity of 0.75, PPV of 24.6%, NPV of 99.8%, and MCC of 0.42 (Table S5). Predicted probabilities were generally higher for EpiHR than for non-EpiHR isolates (Fig. 4B). When stratified by outbreak history, EpiHR isolates from nearly all documented outbreaks (Cooper et al., 2014; Grad et al., 2012; Im et al., 2021; Jenkins et al., 2015; Mellmann et al., 2011; Rusconi et al., 2016) showed predicted probabilities above the default classification threshold, although two isolates were classified as non-EpiHR (Fig. 4C). Threshold-dependent analysis demonstrated that increasing the probability threshold from 0.1 to 0.9 reduced false-positive classifications from 1,182 to 57 and increased PPV from 10.99% to 60.69%, while decreasing true-positive classifications from 146 to 88 (Table S5). Calibration analysis further showed imperfect agreement between predicted probabilities and observed EpiHR frequencies, indicating that model outputs should be interpreted as prioritization scores rather than calibrated probabilities of EpiHR status (Fig. S2).

Performance was further examined according to isolation source, pathotype, serotype, and ST (Table S6). Within the predominant O157:H7 and ST11 subgroups, the model identified all EpiHR isolates, yielding sensitivities of 1.00; however, specificity was markedly reduced in both subgroups, indicating limited discrimination between EpiHR and non-EpiHR isolates within these genomic backgrounds. In contrast, among non-O157:H7 and non-ST11 isolates, sensitivities remained 0.90 and 0.91, respectively, with specificities of 0.79 and 0.80. These findings demonstrate high sensitivity for identifying EpiHR isolates in the independent test dataset, while also indicating limited within-background discrimination among the predominant O157:H7 and ST11 isolates.

### Virulence-associated features contributing to EpiHR classification

To identify genomic features associated with EpiHR classification, feature importance was evaluated using mean absolute SHAP values derived from the selected RF model (Fig. 5). SHAP values quantify the magnitude of each feature’s contribution to model predictions (Wang et al., 2022), with EpiHR designated as the positive class. Among all features, *traT* exhibited the highest mean absolute SHAP value (> 0.03), indicating a strong contribution to model predictions. The *traT* gene encodes an outer membrane protein associated with serum resistance and has been implicated in complement evasion and enhanced bacterial survival in invasive and extraintestinal infections (Achtman et al., 1977; Montenegro et al., 1985). Other virulence-associated genes, including *etpB* and STh/STIb, showed relatively high mean absolute SHAP values. The *etpB* gene encodes an outer membrane transporter of the ETEC two-partner secretion system involved in the secretion of the glycosylated adhesin EtpA (Fleckenstein et al., 2006), whereas STh (*estA*) and STIb (*estB*) encode heat-stable enterotoxins characteristic of ETEC (Wang et al., 2019). The total number of virulence-associated genes detected in each isolate also ranked among the influential features (Fig. 5). The distribution of clusters of orthologous groups (COG) functional categories among the top 50 features is shown in Fig. S3, with a substantial proportion classified in the “Function unknown” category.

### Evaluation of reduced-feature models

Reduced-feature RF models were evaluated using a leakage-controlled analysis (Fig. 6A). The AUC increased from 0.83 with five features to 0.93 with ten features and reached 0.96 and 0.97 with 15 and 20 features, respectively, with little additional improvement thereafter. As ten features represented the smallest model size achieving an AUC above 0.90, this size was selected for the reduced-feature model. When evaluated on the independent test dataset, the reduced-feature model achieved an AUC of 0.79 (Fig. 6B), indicating reduced but measurable discriminatory performance after substantial feature reduction. SHAP analysis of the reduced model showed contributions toward both classification directions (Fig. S4).

## Discussion

The ability to prioritize EpiHR pathogenic *E. coli* isolates has important implications for foodborne outbreak surveillance. In this study, we developed ML models using WGS-derived virulence-associated genomic features to distinguish EpiHR from non-EpiHR pathogenic isolates. Among the four algorithms evaluated, the RF model showed the highest discriminatory performance in cross-validation and retained high sensitivity in the independent test dataset, with an AUC of 0.97 and a sensitivity of 0.98. These findings support the potential utility of this framework for surveillance-oriented prioritization of pathogenic *E. coli* isolates requiring further epidemiological attention.

The phylogenetic and subgroup analyses provide important context for interpreting model performance. In our dataset, isolates sharing similar source, pathotype, serotype, and ST frequently clustered together, suggesting that strains with comparable molecular characteristics tend to harbor similar virulence gene compositions. Prominent EpiHR-associated genomic backgrounds included O157 among EHEC isolates and O159 among ETEC isolates. These backgrounds have also been reported previously (Kim et al., 2017; Lucatelli et al., 2024). LOGO validation showed that discrimination was retained across held-out STs and serotypes, whereas performance decreased more substantially when isolation source was excluded during model training. Consistently, the independent test dataset was strongly enriched for O157 and ST11 within the EpiHR group, and the reduced specificity observed within these predominant subgroups indicated limited discrimination between EpiHR and non-EpiHR isolates sharing these genomic backgrounds. Thus, although the model captured predictive information beyond individual STs or serotypes, source-associated structure contributed to its performance.

Several SHAP-ranked features, including *traT*, *etpB*, STh/STIb, and overall virulence-associated gene burden, are biologically plausible in the context of host interaction and enterotoxin-associated pathogenicity, but their importance should be interpreted as predictive associations rather than as mechanistic determinants of intrinsic virulence. Mean absolute SHAP values reflect the magnitude of feature contributions, not their direction. For example, the presence of *traT* contributed predominantly toward non-EpiHR prediction in the reduced model. In addition, feature importance may partly reflect ST, serotype, or pathotype composition. Therefore, functional validation using epidemiologically diverse isolates will be required to clarify the biological relevance of these predictive features.

Reducing the number of genomic features was intended to explore whether a more parsimonious model could retain useful discrimination while limiting feature complexity. Following leakage-controlled feature-number evaluation, the top ten feature model achieved an AUC of 0.79 in the independent test dataset, substantially lower than that of the full-feature model. This result indicates that a limited subset of predictive features captures part of the EpiHR-associated signal, but additional features contribute meaningfully to overall model performance. Accordingly, the reduced-feature model should be regarded as an exploratory basis for future simplified screening approaches rather than a validated alternative to the full genomic model.

In the independent test dataset, two outbreak-associated food-derived EHEC isolates of serotype O145:H28 were predicted as non-EpiHR despite their documented outbreak association. Most O145:H28 isolates in the test dataset were also assigned to the non-EpiHR category (61/63, 96.8%), suggesting that outbreak-associated isolates within this serotype did not share a uniform virulence-associated genomic profile captured by the model. In addition, the high sensitivity but relatively low PPV and imperfect calibration observed in the test dataset indicate that predicted probabilities are most appropriately interpreted as prioritization scores rather than calibrated estimates of EpiHR status. Collectively, model outputs should be considered together with epidemiological, environmental, and outbreak investigation data.

Previous studies have applied ML approaches to investigate pathogenic or virulence-related traits in *E. coli*, often focusing on specific pathotypes or clinical endpoints using WGS-derived features. For example, Im et al. (2021) developed an SVM-based model using WGS data to discriminate STEC from non-pathogenic isolates, demonstrating high classification accuracy and identifying genes potentially associated with STEC pathogenicity through permutation importance analysis. Other studies have used supervised learning to identify *eae*-positive STEC strains linked to severe disease outcomes or to predict zoonotic and infection potential in O157 isolates based on pangenome features (Lupolova et al., 2016). In contrast to these pathotype-specific approaches, our study developed and externally evaluated an interpretable genomic framework for EpiHR prioritization across multiple pathogenic *E. coli* pathotypes, extending ML-based surveillance beyond a single pathotype.

Although these findings demonstrate the potential utility of our approach, several limitations should be considered. First, EpiHR and non-EpiHR were defined according to clinical origin and documented outbreak association rather than experimentally confirmed intrinsic virulence. Clinical disease and outbreak occurrence may also be affected by exposure, host susceptibility, environmental conditions, and surveillance practices. Second, the independent test dataset was assembled from publicly available data with heterogeneous study objectives and metadata quality, and its EpiHR group was strongly enriched for O157:H7 and ST11 isolates. In addition, because the training dataset was derived from a nationwide surveillance study conducted in South Korea, the transferability of the current framework to other epidemiological settings may be affected by regional differences in circulating pathogenic *E. coli* lineages, exposure patterns, and surveillance practices. Although the additional subgroup and LOGO analyses provided a more transparent assessment of these issues, further evaluation in geographically and phylogenetically diverse datasets is required. Third, the high sensitivity but relatively low PPV observed in the test dataset indicates that positive model predictions should be followed by epidemiological assessment. Fourth, the current model relied primarily on binary presence or absence of curated virulence-associated genes and therefore did not capture allelic variation, subtype-specific functional differences, regulatory alterations, or potentially relevant pseudogene status. Finally, reliance on a curated database may limit identification of newly recognized or incompletely characterized genomic features. Future studies incorporating geographically and phylogenetically diverse isolates, sequence-level feature annotation, and functional validation will be important for improving model robustness and biological interpretability.

In conclusion, this study demonstrates that an ML model based on virulence-associated features can prioritize EpiHR pathogenic *E. coli* isolates. When interpreted together with epidemiological information, the model provides a sensitive and transparent approach for identifying isolates warranting further investigation. This framework offers a practical foundation for genome-informed foodborne outbreak surveillance and, with further validation in broader and more diverse datasets, may support future implementation in routine surveillance settings.

## Figures and Tables

**Fig. 1.**
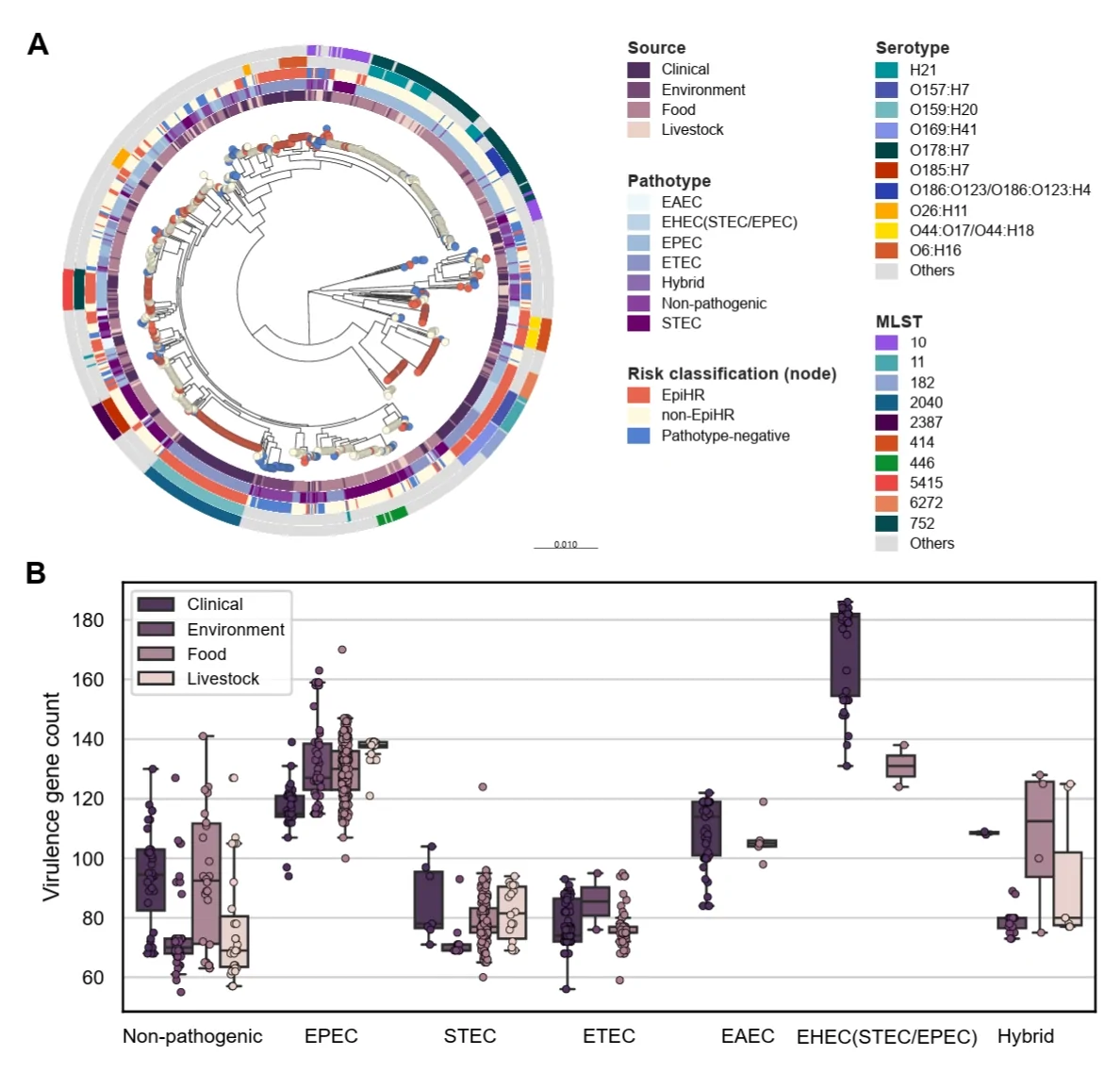
Phylogenetic diversity of *E. coli* isolates and distribution of virulence-associated gene count. (A) Maximum-likelihood tree based on core-genome single-nucleotide polymorphisms illustrating the diversity of the 1,029 *E. coli* isolates analyzed in this study. Node colors indicate risk group classification. The color strips represent isolation source, pathotype, serotype, and sequence type, from the inside out. The ten most frequent sequence types and serotypes are shown, with remaining categories grouped as others. An interactive phylogenetic tree is available at https://microreact.org/project/waCGNP8rxA177yzzZd1trN-figure1a. (B) Distribution of virulence-associated gene counts across pathotypes and isolation sources. Each point represents an individual isolate. Boxplots indicate the interquartile range (IQR), with median values and whiskers extending to 1.5× IQR.

**Fig. 2.**
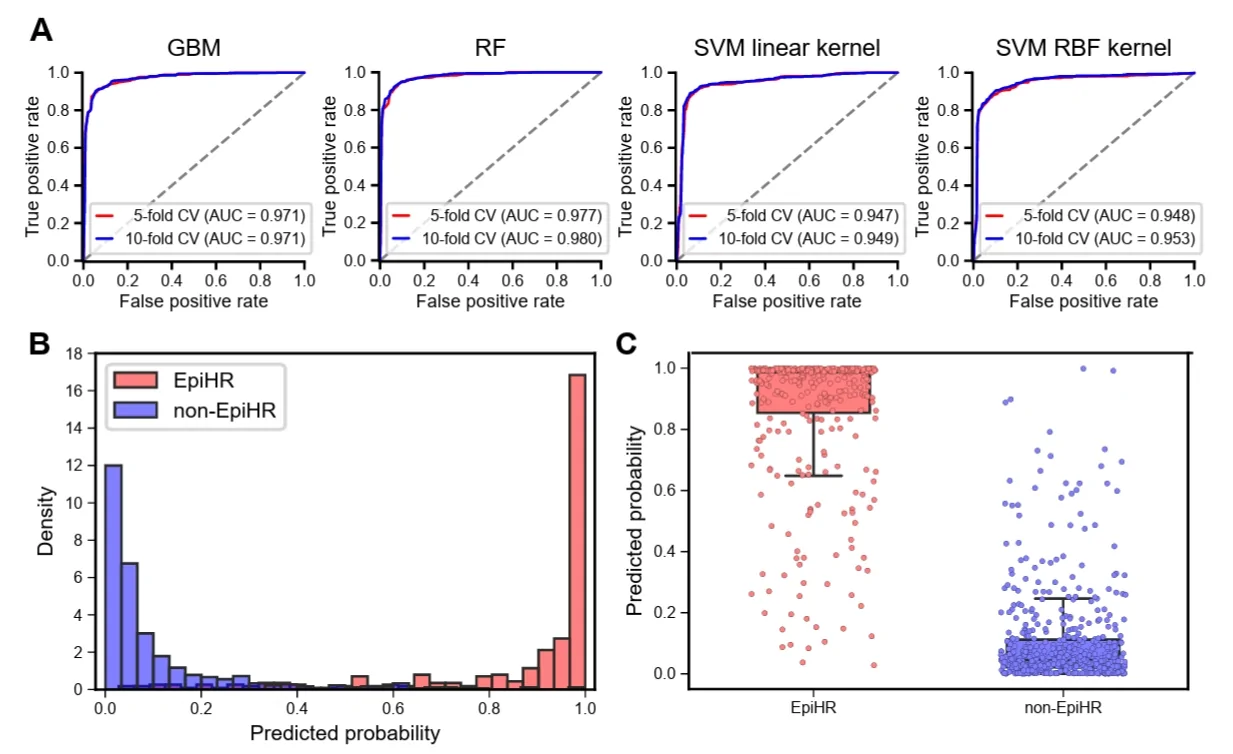
Performance of machine learning models and distribution of predicted probabilities. (A) Receiver operating characteristic curves for four machine learning algorithms evaluated using 5-fold and 10-fold cross-validation. Corresponding area under the curve values are shown. (B) Distribution of predicted probabilities for epidemiologically defined high-risk (EpiHR) based on pooled out-of-fold predictions from the selected random forest model in 10-fold cross-validation. (C) Boxplots comparing predicted probabilities between EpiHR and non-EpiHR isolates. Each point represents an isolate. Boxes indicate the interquartile range (IQR), with median values and whiskers extending to 1.5× the IQR. GBM, gradient boosting machine; RF, random forest; SVM, support vector machine; RBF, radial basis function; CV, cross-validation; AUC, area under the curve.

**Fig. 3.**
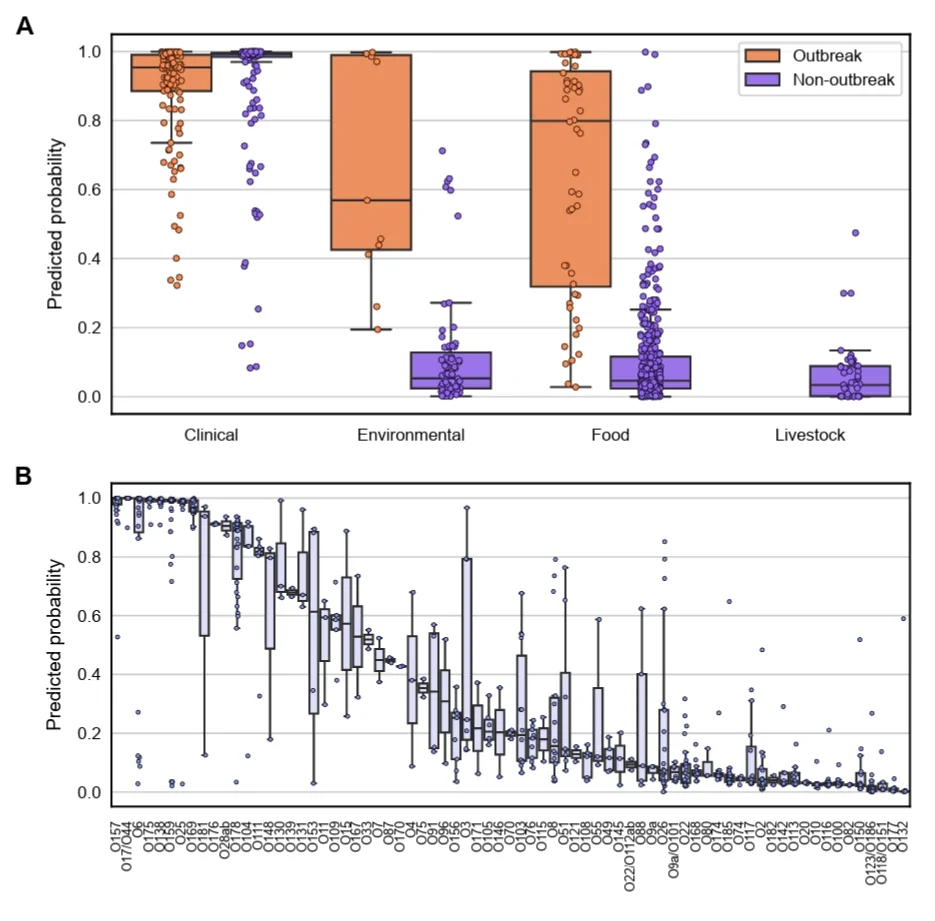
Patterns of predicted probabilities across isolation sources and serotypes. (A) Distribution of predicted epidemiologically defined high-risk (EpiHR) probabilities according to isolation source and documented outbreak association. Pathogenic isolates were stratified as outbreak-associated or non-outbreak within each isolation source. (B) Distribution of predicted EpiHR probabilities across O-serotypes. Serotypes represented by a single isolate were excluded. Boxes indicate the interquartile range (IQR) with median values and whiskers extending to 1.5× the IQR.

**Fig. 4.**
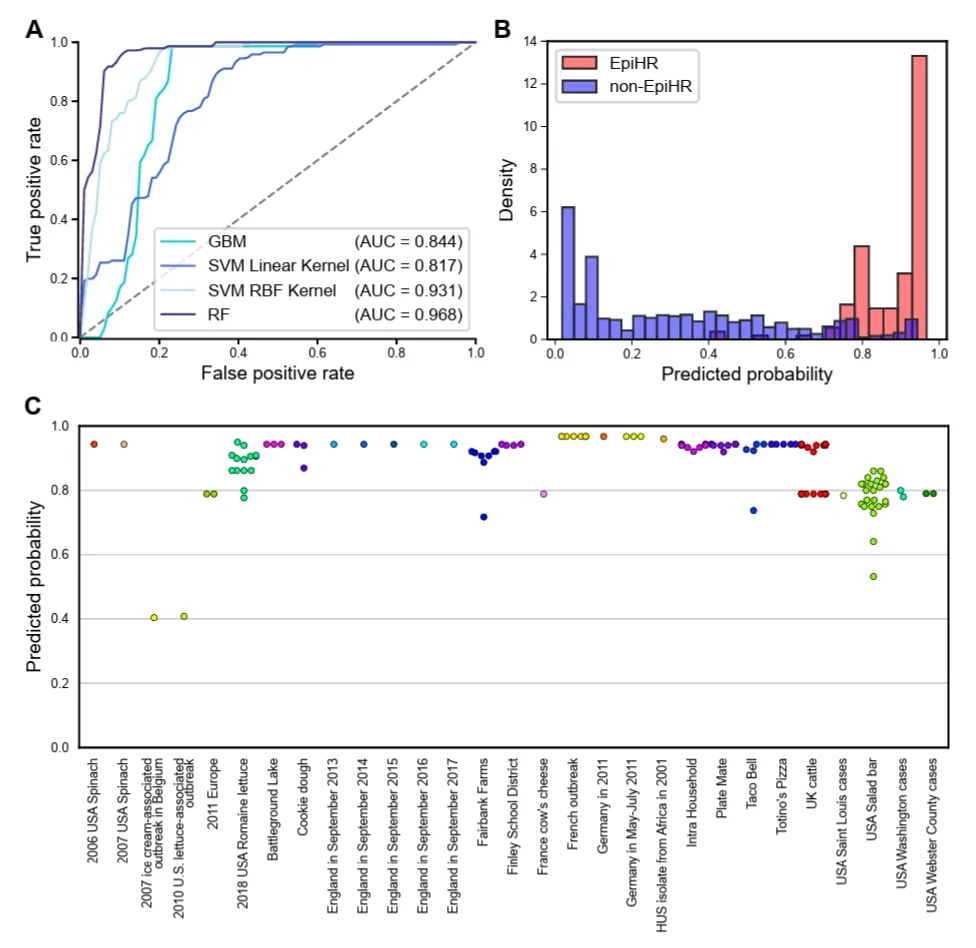
Model performance and predicted probabilities in the independent test dataset. (A) Receiver operating characteristic curves showing the performance of four machine learning algorithms on the test dataset. Corresponding area under the curve values are shown. (B) Distribution of predicted epidemiologically defined high-risk (EpiHR) probabilities for EpiHR and non-EpiHR isolates in the test dataset. (C) Distribution of predicted EpiHR probabilities, all of which were outbreak-associated, stratified by outbreak history. GBM, gradient boosting machine; RF, random forest; SVM, support vector machine; RBF, radial basis function; AUC, area under the curve.

**Fig. 5.**
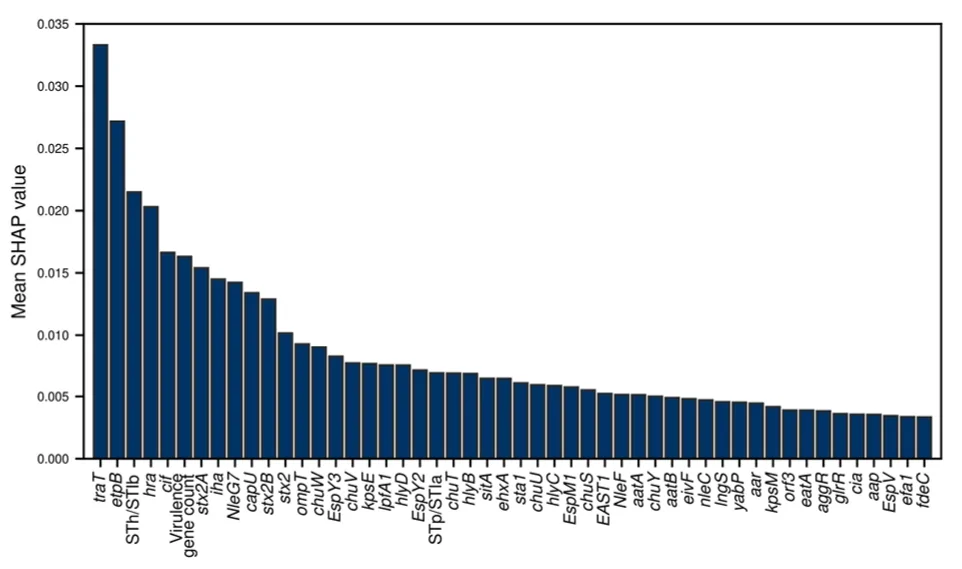
Important features contributing to random forest predictions. Bar plot showing the top 50 features by mean absolute Shapley additive explanations (SHAP) values in the random forest model. Higher SHAP values indicate greater contribution of a feature to model predictions.

**Fig. 6.**
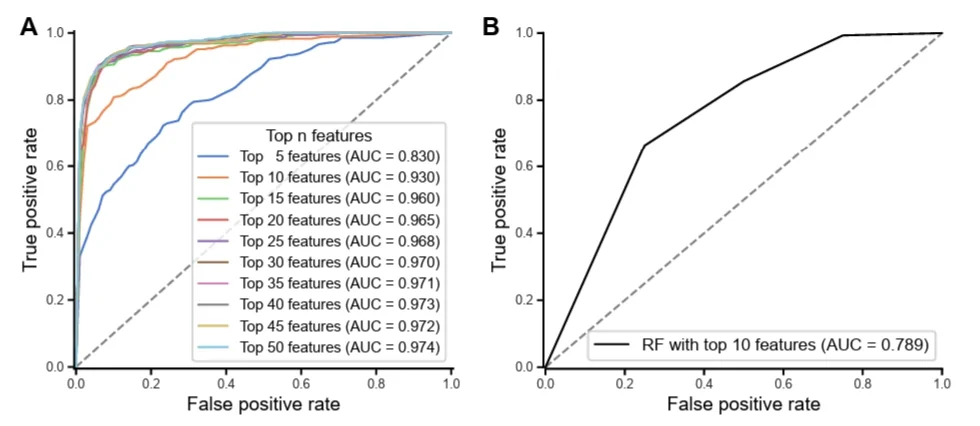
Performance of random forest models with incremental feature sets and external validation. (A) Receiver operating characteristic (ROC) curves of random forest (RF) models constructed using the top N features ranked by mean absolute Shapley additive explanations values, added in increments of five. Model performance was evaluated using 10-fold cross-validation. (B) ROC curve of the RF model using the top ten selected features evaluated on the independent test dataset. AUC, area under the curve.

**Table 1.** Out-of-fold performance metrics of four machine learning algorithms evaluated using cross-validation

Algorithm	CV scheme	Accuracy	Precision	Sensitivity	Specificity	F1-score	MCC	AUC
GBM	5-fold	0.926	0.915	0.894	0.946	0.905	0.844	0.971
	10-fold	0.929	0.923	0.894	0.952	0.909	0.851	0.971
RF	5-fold	0.929	0.926	0.891	0.954	0.908	0.851	0.977
	10-fold	0.933	0.927	0.900	0.954	0.913	0.858	0.980
SVM (linear kernel)	5-fold	0.911	0.891	0.883	0.930	0.887	0.814	0.947
	10-fold	0.921	0.909	0.889	0.943	0.899	0.835	0.949
SVM (RBF kernel)	5-fold	0.903	0.898	0.851	0.937	0.874	0.796	0.948
	10-fold	0.907	0.906	0.851	0.943	0.878	0.803	0.953

GBM, gradient boosting machine; RF, random forest; SVM, support vector machine; RBF, radial basis function; CV, cross-validation; MCC, Matthews correlation coefficient; AUC, area under the curve. EpiHR isolates were designated as the positive class.

## Supplementary Information

Table S1. Overview of training datasets

Table S2. Overview of the independent test dataset

Table S3. Fold-level performance of the random forest model in 10-fold cross-validation of the training dataset

Table S4. Performance of the random forest model in leave-one-group-out cross-validation of the training dataset

Table S5. Threshold-dependent performance of the random forest model in the independent test dataset

Table S6. Stratified performance of the random forest model in the independent test dataset

Fig. S1. Workflow of the random forest-based risk-prioritization pipeline for pathogenic *E. coli*.

Fig. S2. Calibration of the random forest model in the independent test dataset.

Fig. S3. Cluster of orthologous groups functional categories of the top 50 important features.

Fig. S4. SHAP dot plot of the top ten features in the reduced random forest model.
