# Supplementary material for: Genomic signatures associated with epidemiologically defined high-risk pathogenic Escherichia coli isolates identified by interpretable machine learning
Source: J Microbiol. 2026 Aug 31;64(8):e2604011. doi: 10.71150/jm.2604011 (PMC13688133; doi:10.71150/jm.2604011)
Supplement: Supplementary file 1 [file jm-2604011-Supplementary-Fig-S1.pdf]

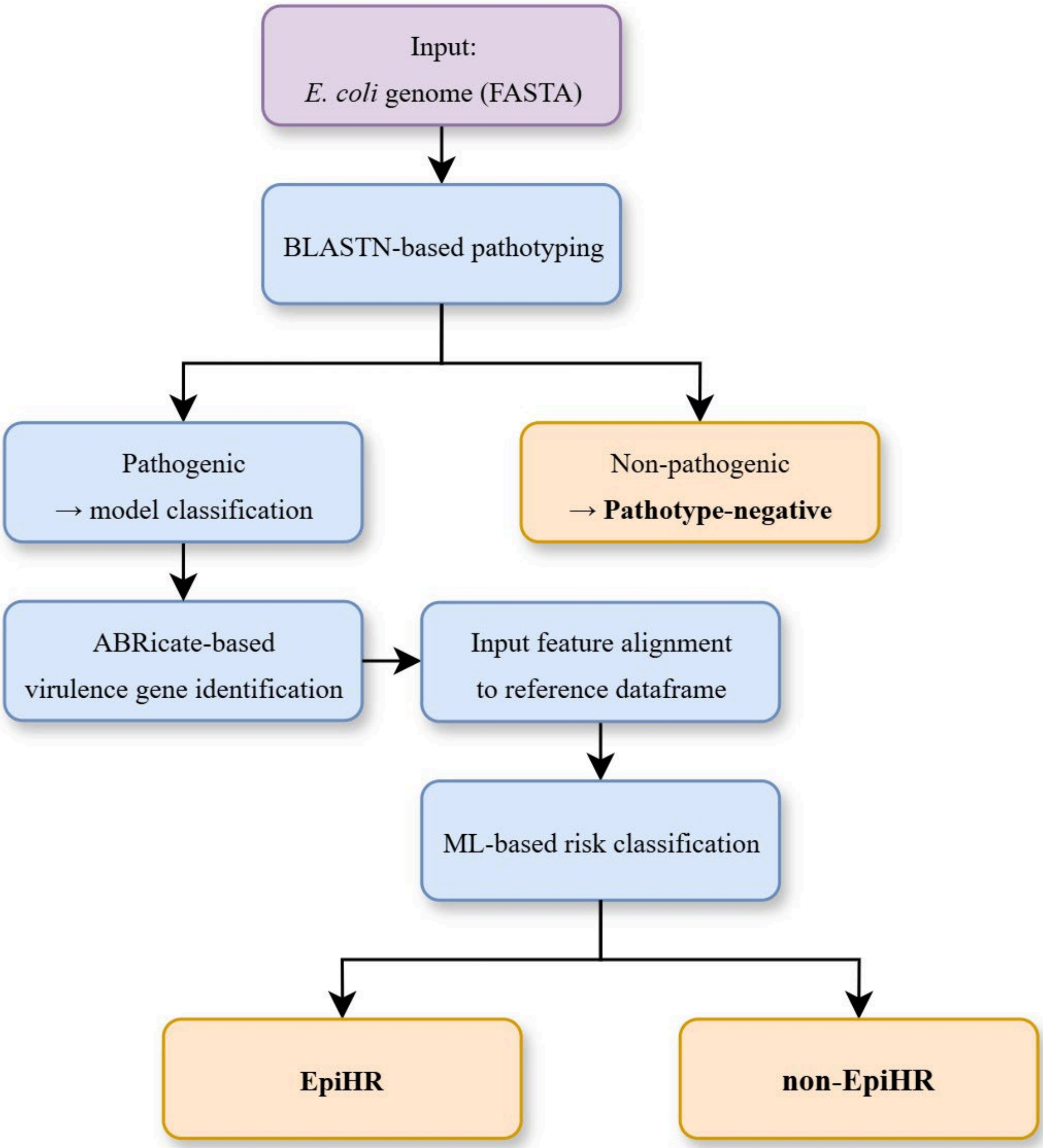

Fig. S1. Workflow of the random forest-based risk-prioritization pipeline for pathogenic *E. coli*. Draft *E. coli* genome assemblies in FASTA format are subjected to BLASTN-based pathotyping. Isolates assigned as non-pathogenic by pathotyping are reported as pathotype-negative and excluded from random forest-based epidemiologically defined high-risk (EpiHR) classification, whereas isolates assigned to pathogenic pathotype undergo ABRicate-based virulence-associated gene identification and feature alignment. The random forest model then estimates EpiHR probability and classifies isolates as EpiHR or non-EpiHR.
