# Supplementary material for: Genomic signatures associated with epidemiologically defined high-risk pathogenic Escherichia coli isolates identified by interpretable machine learning
Source: J Microbiol. 2026 Aug 31;64(8):e2604011. doi: 10.71150/jm.2604011 (PMC13688133; doi:10.71150/jm.2604011)
Supplement: Supplementary file 2 [file jm-2604011-Supplementary-Fig-S2.pdf]

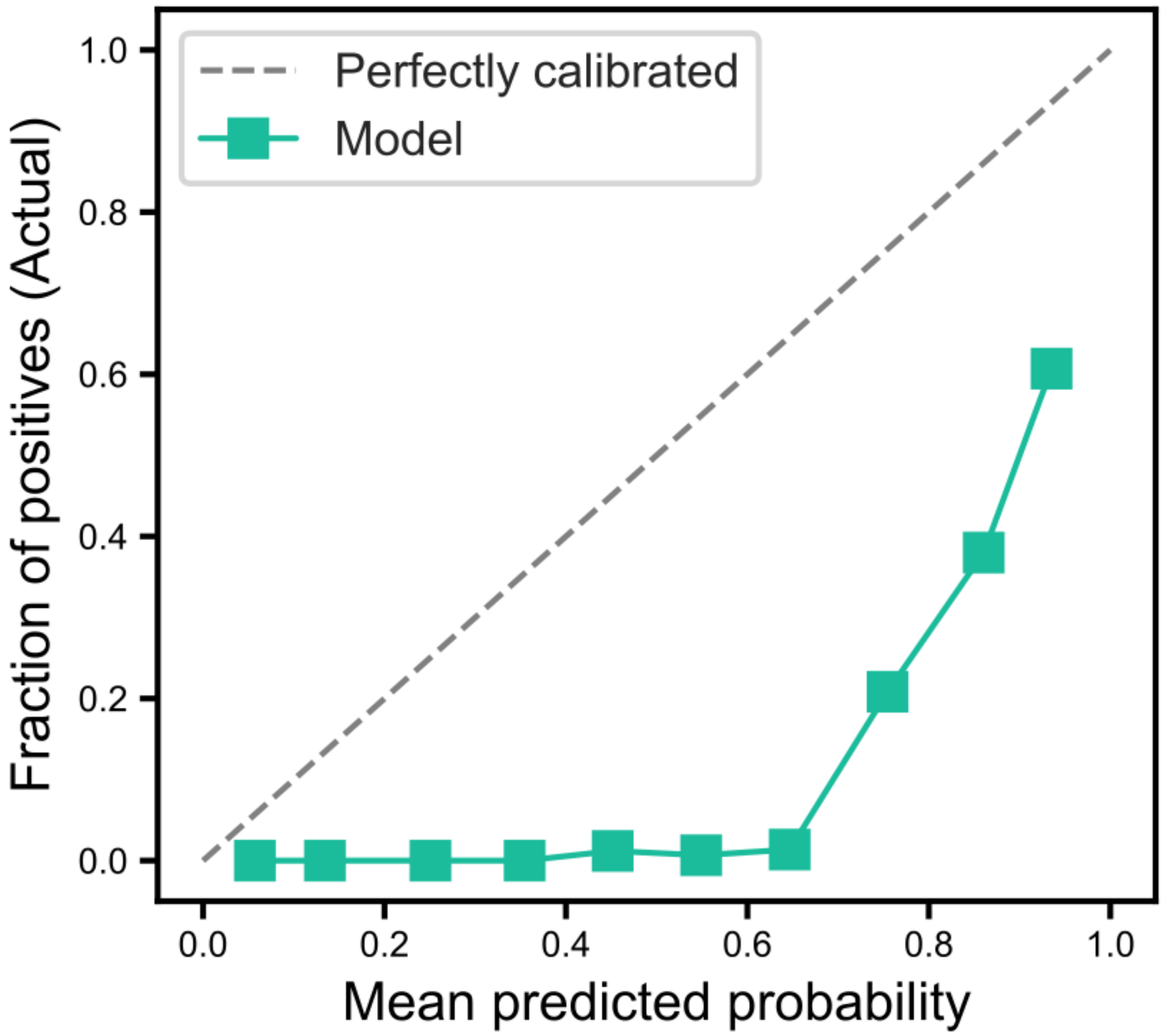

Fig. S2. Calibration of the random forest model in the independent test dataset. Calibration curve comparing mean predicted epidemiologically defined high-risk (EpiHR) probabilities with the observed fraction of EpiHR isolates in the independent test dataset. The dashed diagonal line indicates perfect calibration, and the solid line with square markers represents the calibration of the selected random forest model. EpiHR isolates were designated as the positive class.
