# Supplementary material for: Genomic signatures associated with epidemiologically defined high-risk pathogenic Escherichia coli isolates identified by interpretable machine learning
Source: J Microbiol. 2026 Aug 31;64(8):e2604011. doi: 10.71150/jm.2604011 (PMC13688133; doi:10.71150/jm.2604011)
Supplement: Supplementary file 3 [file jm-2604011-Supplementary-Fig-S3.pdf]

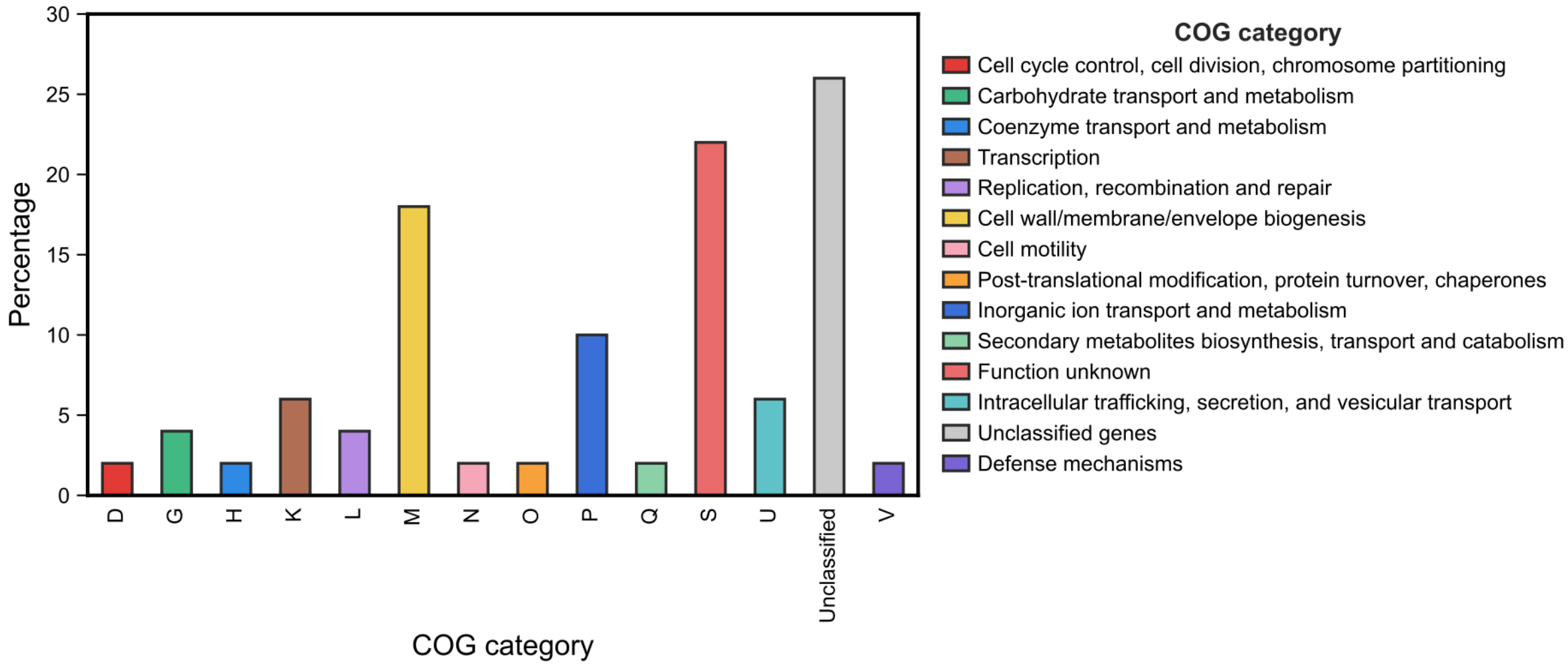

Fig. S3. Cluster of orthologous groups functional categories of the top 50 important features. Distribution of clusters of orthologous groups (COG) functional categories among the top 50 features ranked by mean absolute Shapley additive explanations (SHAP) values in the random forest model. Percentages represent the proportion of genes assigned to each category. Features without COG annotation are shown as unclassified.
