# Supplementary material for: Genomic signatures associated with epidemiologically defined high-risk pathogenic Escherichia coli isolates identified by interpretable machine learning
Source: J Microbiol. 2026 Aug 31;64(8):e2604011. doi: 10.71150/jm.2604011 (PMC13688133; doi:10.71150/jm.2604011)
Supplement: Supplementary file 4 [file jm-2604011-Supplementary-Fig-S4.pdf]

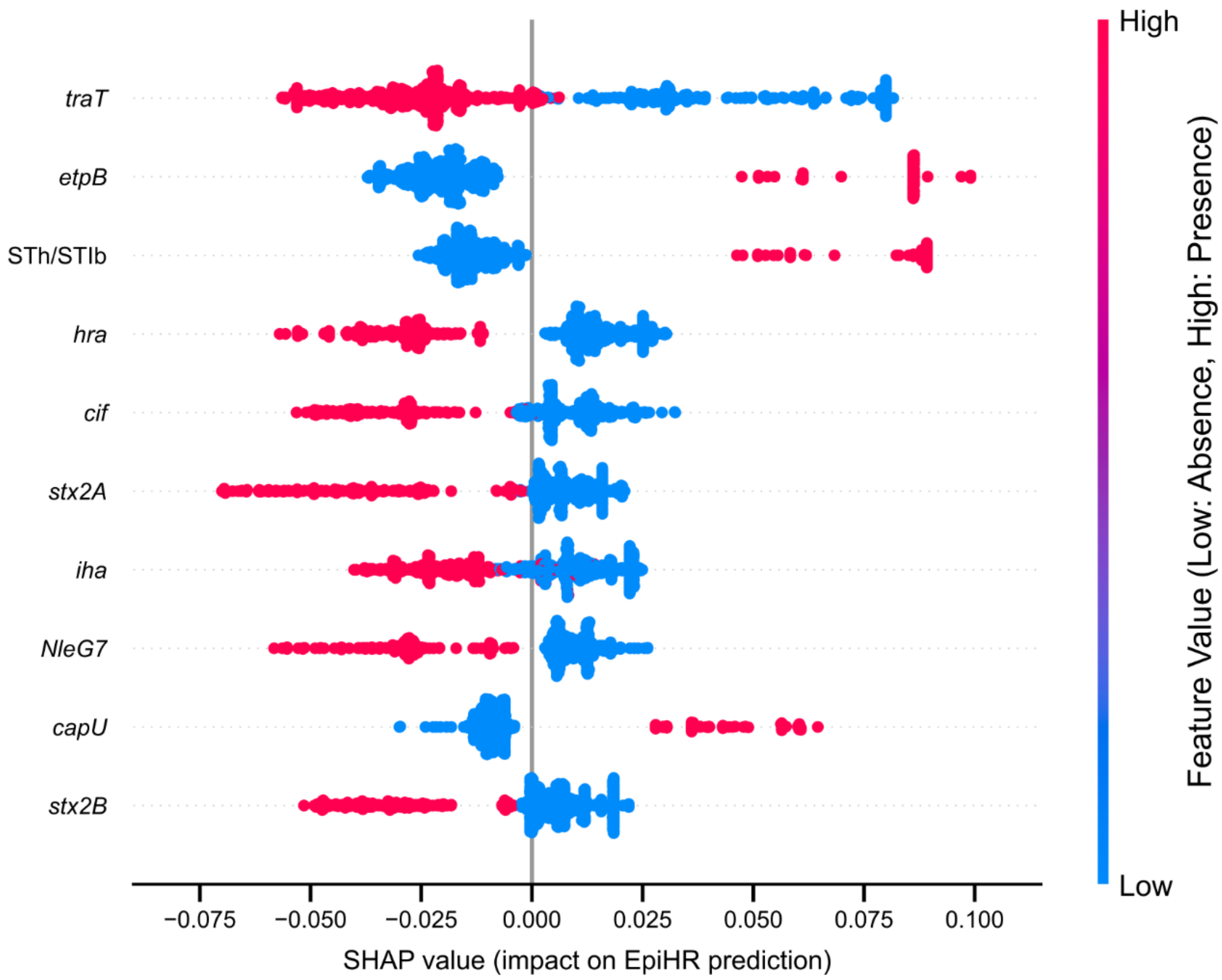

Fig. S4. SHAP dot plot of the top ten features in the reduced random forest model. Shapley additive explanations (SHAP) dot plot showing the contribution of the ten most important features to model predictions. Each dot represents an individual isolate, with the X-axis indicating the SHAP value and colors representing binary feature values, where low values indicate gene absence and high values indicate gene presence. Positive SHAP values indicate contributions toward epidemiologically defined high-risk (EpiHR) prediction, whereas negative values indicate contributions toward non-EpiHR prediction.
